# Using a Semiautomated Procedure (CleanADHdata.R Script) to Clean Electronic Adherence Monitoring Data: Tutorial

**DOI:** 10.2196/51013

**Published:** 2024-05-22

**Authors:** Carole Bandiera, Jérôme Pasquier, Isabella Locatelli, Marie P Schneider

**Affiliations:** 1 School of Pharmaceutical Sciences University of Geneva Geneva Switzerland; 2 Institute of Pharmaceutical Sciences of Western Switzerland University of Geneva Geneva Switzerland; 3 Center for Primary Care and Public Health University of Lausanne Lausanne Switzerland

**Keywords:** medication adherence, digital technology, digital pharmacy, electronic adherence monitoring, data management, data cleaning, research methodology, algorithms, R, semiautomated, code, coding, computer science, computer programming, medications, computer script

## Abstract

**Background:**

Patient adherence to medications can be assessed using interactive digital health technologies such as electronic monitors (EMs). Changes in treatment regimens and deviations from EM use over time must be characterized to establish the actual level of medication adherence.

**Objective:**

We developed the computer script CleanADHdata.R to clean raw EM adherence data, and this tutorial is a guide for users.

**Methods:**

In addition to raw EM data, we collected adherence start and stop monitoring dates and identified the prescribed regimens, the expected number of EM openings per day based on the prescribed regimen, EM use deviations, and patients’ demographic data. The script formats the data longitudinally and calculates each day’s medication implementation.

**Results:**

We provided a simulated data set for 10 patients, for which 15 EMs were used over a median period of 187 (IQR 135-342) days. The median patient implementation before and after EM raw data cleaning was 83.3% (IQR 71.5%-93.9%) and 97.3% (IQR 95.8%-97.6%), respectively (Δ+14%). This difference is substantial enough to consider EM data cleaning to be capable of avoiding data misinterpretation and providing a cleaned data set for the adherence analysis in terms of implementation and persistence.

**Conclusions:**

The CleanADHdata.R script is a semiautomated procedure that increases standardization and reproducibility. This script has broader applicability within the realm of digital health, as it can be used to clean adherence data collected with diverse digital technologies.

## Introduction

Patient adherence to medication may be suboptimal, as it is estimated that only 50% to 70% of patients take their medications regularly as prescribed [1]. Medication nonadherence leads to poor clinical outcomes [1-3], additional hospitalizations [4], and increased health care costs [1]. Medication adherence is defined as the extent to which a patient takes the medication as prescribed by the provider, ideally based on a shared decision-making process. It is characterized by the initiation (ie, the patient takes the first dose), implementation (ie, the extent to which the patient takes the prescribed regimen according to the recommended dose, timing, and regimen), and discontinuation (ie, the patient stops taking the treatment earlier than planned with the prescriber) of the medication [5]. Treatment persistence is characterized by the time elapsed between initiation and discontinuation. Medication adherence can be assessed through measurement methods that are subjective (ie, patient self-report questionnaires) or objective (ie, medication refill and interactive digital technology such as electronic monitors [EMs]) [6,7].

The EM is an interactive digital health technology, considered a gold standard for measuring medication implementation [8,9]. The medication event monitoring system (MEMS; MEMS and MEMS Adherence Software, AARDEX Group) is an example of an EM. It is a pill bottle with a chip located in the EM cap, registering the date and time of each EM opening, which is considered a proxy for the date and time of each consecutive medication intake. A MEMS pill bottle contains medication, and a patient can use one or several MEMS at a time. Despite the advantages of using EM (ie, providing longitudinal patterns of adherence over time and providing electronic adherence feedback to the patient in interventions aimed at improving adherence [10]), some methodological challenges must be considered when analyzing EM data. Indeed, EM can be misused (ie, the patient can open the EM without swallowing the drug, known as curiosity checks, and the patient can take the drug without opening the EM, known as pocket doses), or the patient may not use the EM during specific periods (eg, during holidays or hospitalizations). In addition, the patient’s treatment regimen can be modified during the monitoring period, leading to changes in the dose or regimen; temporary interruptions of the treatment and alternative medication may be prescribed [11]. If these events are not rigorously collected and reported in EM raw data sets before analysis, patients’ actual level of adherence tends to be underestimated [12]. Thus, data cleaning improves the accuracy of the EM raw data set before analysis and decreases data misinterpretation.

In the past decade, digital technologies—such as mobile apps, EMs, ingestible sensors, weekly electronic pillboxes, blister pack technologies, electronic patient self-report diaries, or insurance claim databases [13]—have been increasingly used to measure real-time components of adherence (ie, initiation, implementation, and persistence [5]). Digital health innovations and technologies can be used to contribute and facilitate interventions aiming at supporting medication adherence [14]. In parallel, the quality of adherence data analysis has increased due to guidelines on operational adherence definitions [15], advanced statistical analysis [16,17], and semiautomated procedures to analyze adherence in health care databases (eg, the *Adhere-R* package [18]). However, to date, the research community lacks guidance on the methodology to collect, manage, clean, and analyze adherence electronic monitoring database, whereas it is known that heterogeneous data management impacts adherence outcomes [19].

Some authors have attempted to provide guidelines to manually clean EM adherence data sets, such as inserting pocket doses, deleting excessive EM openings, and censoring the EM data in periods during which the patient did not use the EM [19-21]. Rotzinger et al [22] presented a step-by-step procedure to manually clean EM adherence data sets based on pill-count data when available and proposed to manually add pocket doses and nonmonitored periods if they were validated. However, manual EM data cleaning is prone to errors, lacks reliability, and is time-consuming. In addition, enriching the EM data set with relevant covariables (eg, demographic, clinical, or medication adherence–related data) would allow in-depth adherence analysis. To date, no semiautomated or automated procedure exists in the literature to rigorously clean and enrich EM adherence data sets prior to analysis.

We present an open-source script developed in the statistical software R (R Foundation for Statistical Computing), named CleanADHdata.R and available at GitHub [23], to clean and enrich EM raw data sets before implementation and persistence analysis. The CleanADHdata.R script was developed to clean and enrich EM adherence raw data for cyclic or continuous regimens prescribed for solid cancers, collected as part of the optimizing targeted anticancer therapies (OpTAT) study [24]. As this script is intended to be used by researchers to clean and enrich EM adherence data from other studies, this tutorial aims to guide the script users.

## Methods

### Ethical Considerations

The OpTAT study was approved by the local ethics committee “Commission cantonale d’éthique de la recherche sur l’être humain” in 2015 (ID 65/15) and was conducted in accordance with the Declaration of Helsinki principles. All patients signed an informed and written consent form prior to participation for their data to be used in this research [24]. The prospective EM adherence data collection as part of the OpTAT study started in July 2015 and lasted until May 2022 [24]. We developed this tutorial with fully fictitious EM data, blurred with the procedure detailed in the paper, that is, without any information that could identify individual participants.

### EM Raw Data Extraction in the Required Format to Use CleanADHdata.R

To prepare the EM adherence data set for cleaning and enrichment, patient EM raw data were extracted into a text as a CSV or Microsoft Excel (Microsoft Corp) file using dedicated software (MEMS Adherence Software). Three columns entitled *PatientCode*, *Monitor*, and *Date* were indicated in the first row in the required format to be read by the CleanADHdata.R script. The data in columns *PatientCode* and *Monitor* must be in an alphanumeric format, and the data in column *Date* must be in a date-time format or a well-formatted date character string. The filenames of patients’ EM raw data must contain the “eventslist” character string, and the files are to be registered all together in the same file folder (1 EM raw data file can contain the EM data of several patients). The “dailyadherence” format (ie, number of EM openings at each day) is accepted, but an additional column entitled *RecordedOpenings* needs to be added. To allow users to practice using the script, we provided blurred EM raw data sets for 10 patients, available on the web at GitHub [23].

### Choosing the Relevant Variables to Collect for Cleaning and Enriching EM Raw Data

The level of accuracy of the EM data cleaning and enrichment must be adapted to the sample size of each study, its design, and the treatment monitored. It may not be relevant to insert pocket doses if their occurrence represents less than 5% of all doses in a monitored period or if the sample size is very large, that is, >500 patients (estimation), as their impact on adherence outcomes is limited.

Investigators need to choose in advance which relevant variables to collect to clean and enrich the EM data set (eg, regimen changes, hospitalization dates, periods of EM nonuse, and pocket doses), depending on the operational definition of adherence [15], the planned statistical analysis, and the information sources available (eg, patient-reported outcomes or experiences and medical, pharmaceutical, and administrative databases). Whenever possible, 2 different sources should be consulted to confirm the medication regimen and obtain the best possible medication history [25]. In case of discordant information between different sources, investigators must establish a procedure and document the information in a reproducible and reliable way.

In the OpTAT study, the variables chosen were the dates of treatment interruption due to side effects, toxicity, and hospitalization dates. We also collected the side effects and their grades at each medical visit, the changes in the oral anticancer treatment (OAT) regimen and doses, and the discrepancies in EM use reported by patients (eg, periods of EM nonuse during holidays, nonmonitored periods in case of hospitalization, when the patient did not use the EM for any documented reason, or taking pocket doses).

“Timing pocket dosing” is defined as when the patient takes one or several doses out of the EM in advance to be swallowed later the same day (ie, day *x*). Timing pocket dosing matters only if timing adherence is considered in the analysis. When 1 or several doses are prepared in advance to be taken on the day after (ie, day *x*+1 and 24 hours later), we consider the days without any EM openings as a period of EM nonuse, for example, a nonmonitored period. Extra EM openings (eg, by a physician, pharmacist, or patient to check the number of pills left) could also be reported whenever necessary. To insert pocket doses, delete extra EM openings, or set a nonmonitored period, the EM deviations must be carefully documented and reported by the patient before showing him or her the electronic monitoring adherence feedback and must be validated by pill count.

### Gathering the Relevant Variables Into a Predefined Excel File Format

The collected variables described earlier must be gathered into an Excel file called Auxiliary Data using a predefined format. This Excel file is composed of 7 different sheets: *EMInfo* and *Regimen* are sheets to be filled to run the script (Table 1), whereas *PatientCovariables*, *EMCovariables*, *AddedOpenings*, *NonMonitoredPeriods*, and *AdverseEvents* are optional sheets (Multimedia Appendix 1).

In each sheet, the first row contains predefined variable names (eg, *PatientCode*, *Monitor*, and *ExpectedOpenings*; Table 1 and Multimedia Appendix 1). Except for the sheets *PatientCovariables* and *EMCovariables*, if other variables are inserted, they will be ignored by the R script (eg, the column *Comments* can be added in the different sheets to help the investigator during data preparation in the Auxiliary Data file). To note, all variable names are case-sensitive.

An example of an Auxiliary Data Excel file is available on the web at GitHub [23]; the variables inserted will allow cleaning and enriching the blurred EM raw data set.

**Table 1:** Description of the mandatory sheets to be filled in the Auxiliary Data Excel file.

Sheets	Mandatory variables in the Excel file, each of them filled in separate sheets	
	EMInfo	Regimen
Description of each variable as a header of a row to be filled in the required format	PatientCode is the identifier of the patient. Monitor is the identifier of each EM^a^ used by the patient (1 EM per line). StartDate: For each EM, start date of use. EndDate: For each EM, end date of use.	PatientCode is the patient’s identifier. Monitor is the identifier of each EM used by the patient (1 EM per line). ExpectedOpenings (nonnegative integer) is the number of expected EM openings per day based on the prescribed treatment regimen in the period specified between StartDate and EndDate. For each regimen change, a new line is created, associated with the period for which the regimen applies. StartDate: Start date of the period for which the number of daily expected opening applies. EndDate: End date of the period for which the number of daily expected opening applies. On (positive integer): In case of a cyclic regimen, the number of days during the On phase in which at least 1 EM opening is to be expected. This variable must be left blank in case of a continuous regimen. Off (positive integer): In case of a cyclic regimen, the number of days during the Off phase in which the treatment is paused. This variable must be left blank in case of a continuous regimen.
Comments	The cleaned data set is truncated by the start and end date of each EM use.	On and Off variables: The cycle begins with the number of expected openings during the consecutive days of the On variable and is followed by 0 expected openings during the number of days of the Off variable. If both columns On and Off are empty, the R script will consider a continuous daily regimen. The R script will report an error if only one of the variables is filled.

^a^EM: electronic monitor.

### Using the CleanADHdata.R Script

Once the raw data have been extracted and the Auxiliary Data Excel file has been completed, the CleanADHdata.R script can be executed. The CleanADHdata.R script is available on the web at GitHub [23].

When the script is executed in the statistical software R, a window allows the selection of the Auxiliary Data Excel file. Once the latter has been selected, a second window opens to select the EM raw data folder. After processing, the R script generates the cleaned and enriched EM data set, called implementation.xlsx, with 2 log files located in the same file folder as the Auxiliary Data Excel file.

The log file entitled errors.log will show critical errors that will lead to a wrong implementation calculation (eg, dates inserted with the wrong format or missing data in the Auxiliary Data Excel file). The log file entitled warnings.log indicates certain inconsistencies in the Auxiliary Data file. They do not necessarily imply miscalculation of the implementation but should be reviewed by the investigator (eg, no data were found for a patient who appears in the Auxiliary Data file). Based on the errors and warnings listed, the investigator can correct the content of the Auxiliary Data file or EM raw data file. After all errors and warnings are resolved, the cleaned and enriched EM data set presented in implementation.xlsx is ready for analysis.

The implementation file was divided into 4 sheets: by monitor, by patient, summary by monitor, and summary by patient. See GitHub [23] for the presentation of the cleaned and enriched EM data set (ie, implementation.xlsx file) obtained using CleanADHdata.R. Multimedia Appendix 2 provides the codebook of the Excel implementation file.

### Operational Definition of Implementation

The operational calculation for medication implementation can be found in the *Implementation Calculation* section in the script CleanADHdata.R. On the first sheet by monitor, the medication implementation for the monitor is considered optimal (1) on each day if the number of corrected EM openings is at least equal to the number of expected EM openings. Otherwise, the implementation is suboptimal (0). In the case of a nonmonitored period on day *x*, implementation is not calculated on day *x* (ie, depicted with an empty cell), as recommended in the literature [9].

In the second sheet by patient, the medication implementation for the patient was calculated each day by the product of the implementation outcome of each patient’s EM. In the case of suboptimal implementation (0) at a given day *x* for at least 1 EM monitored, the patient implementation on that day *x* will be suboptimal (0). In the case of a nonmonitored period at a given day *x* for at least 1 EM monitored, the patient implementation on that day *x* will not be calculated and will be considered a nonmonitored day (ie, depicted with an empty cell).

In the third and fourth sheets, medication implementation was presented as a summary by monitor and a summary by patient. In the summary by monitor, the implementation rate is calculated based on the number of days with an optimal implementation (1) for the EM over the number of monitored days for each EM (ie, nonmonitored periods are excluded). In the summary by patient, the implementation rate is calculated based on the number of days with an optimal implementation for all EM used by the patient (1) over the number of monitored days (ie, nonmonitored periods are excluded). Multimedia Appendix 3 summarizes the methodology to use the script through a presentation given at the Adherence Data Analysis workshop during the International Conference for Medication Adherence (ESPACOMP) in November 2022.

## Results

We used CleanADHdata.R to clean the blurred EM raw data of 10 patients according to variables collected in the Auxiliary Data file to obtain the cleaned and enriched EM data set entitled implementation.xlsx. The EM raw data of 10 patients included in OpTAT were made fictitious by the following blurring method: (1) the implementation.xlsx file was scrambled with an R script (ie, age, gender, side effects, length of monitoring periods, and implementation outcomes were mixed up), generating implementation2.xlsx; (2) an Auxiliary Data file was built based on implementation2.xlsx, and variables were randomly modified (eg, periods of monitoring, date of EM openings, dates of nonmonitored periods, number of added openings, regimens, side effects, patient identifier, and EM number were all blurred); and (3) based on this auxiliary data file, the CleanADHdata.R script was executed to generate implementation3.xlsx. The EM raw data were built based on implementation3.xlsx.

Fictitious patient characteristics were inserted in the *PatientCovariables* sheet of the Auxiliary Data file; the median age was 63 (IQR 50-68) years, 4 (40%) patients were women, and 1 (10%) patient discontinued the treatment.

The OAT International Nonproprietary Names and the type of regimen (ie, continuous or cyclic) were introduced in the *EMCovariables* sheet. A total of 8 OATs (pazopanib, trametinib, dabrafenib, palbociclib, everolimus, alectinib, imatinib, and erlotinib) were prescribed. Among them, palbociclib was prescribed with a cyclic regimen scheme (ie, 21 days of treatment followed by a treatment interruption of 7 days), and imatinib was prescribed with an alternate off-label cyclic regimen (ie, 2 different imatinib dosage strengths delivered in 2 EM are prescribed alternatively, 1 day over 2). Patients B, C, H, I, and J used 2 EMs. In total, 15 different EMs were used over a median period of 187 (IQR 135-342) days. Fatigue was the most reported adverse event, mostly reported as grade 1.

Median patient implementation using the blurred EM raw data set before and after cleaning with CleanADHdata.R was 83.3% (IQR 71.5%-93.9%) and 97.3% (IQR 95.8%-97.6%), respectively (Δ+14%). Implementation was <90% for 1 (7%) of 15 EM monitored, and 1 (10%) of 10 patients had an implementation of <90% over the monitored period.

## Discussion

### Main Results

This tutorial presents a semiautomated procedure to clean and enrich raw EM adherence data by using the open-source CleanADHdata.R script to provide a data set ready for analysis along with an initial overview of implementation results at both the EM and patient levels. First, EM raw data were extracted in a predefined format. Then, an Excel sheet was filled in the predefined format to clean and enrich the EM data set with the following mandatory variables: start and end dates of each EM used by each patient and the expected number of daily EM openings during the monitored periods. The investigators then need to define relevant variables that will allow cleaning and enrichment of the EM data set according to the study aims and procedures. These variables were collected from several reliable sources and inserted in the optional Excel sheet in the predefined format. The R script uses both raw data and the Excel file completed with the variables to provide the cleaned and enriched EM data set, entitled implementation.xlsx file. The difference in absolute implementation (Δ+14%) between the raw and cleaned EM data sets is sufficient to consider systematic EM data cleaning in all studies planning to monitor adherence with EM to avoid data misinterpretation.

### Future Developments of a R Script to Analyze EM Data

Automated procedures to prepare and analyze large data sets are increasing and made accessible to the research community, particularly through the interdisciplinary collaborations of health care researchers, statisticians, and computer scientists. Seeking EM adherence data cleaning, we developed an R script to standardize the EM data analysis. Based on the cleaned adherence data set, developing a new R script will allow using the Kaplan-Meier curves for persistence analysis and the generalized estimated equation model for implementation and adherence analysis. The possibility of analyzing adherence in the presence of censoring times to avoid biased results has been recently developed and will be included in the future script [16].

### Strengths, Challenges, and Limitations

Using CleanADHdata.R allows researchers to achieve reliable and reproducible raw EM data cleaning. Standardizing and describing the procedures for EM data set preparation are necessary for adherence research to allow comparison of results using a homogeneous method across studies. The script CleanADHdata.R can be used to clean adherence data collected with the EM and other digital technologies and can be adapted according to the study aims and designs (eg, the calculation of treatment implementation can be revised in the dedicated coding section). Providing blurred data sets, we allow users to practice using CleanADHdata.R. Moreover, users can improve the script by providing feedback directly on the GitHub platform so it can be updated upon needs.

When using CleanADHdata.R, the investigator needs to carefully fill the variables in an Excel file in a predefined format, which requires time and resources. We recommend collecting these data and filling the Excel file progressively at each patient interview, new prescription, and notification in the medical record. Defining patients’ daily expected EM openings based on drug regimen information from several sources (eg, providers, patients, caregivers, and electronic medical and pharmaceutical files) can be challenging, as discrepancies can occur across sources. The investigator needs to define the most reliable information to be collected according to the medication adherence components definition. To ensure consistency across adherence studies, these decisions need to be documented and depicted in the methodology of the publications.

Our blurred data set provides EM data for a limited number of patients (n=10), a small sample size that only estimates the difference between implementation according to raw and clean data sets. However, using real data from 58 EMs used by 40 patients included in the OpTAT study, a significant difference in implementation was confirmed using the raw and clean data sets (69% vs 92%, +Δ23%) [26]. More studies with larger sample sizes are needed to explore the differences in implementation outcomes between raw and clean data sets.

### Conclusions

Developing a semiautomated procedure to clean and enrich EM adherence data sets saves time, decreases data-handling errors, and increases reproducibility. Investigators and statisticians worked together to develop the open-source CleanADHdata.R script and described its use in this tutorial to guide future users. Consistent use of this script across research teams will increase the quality and standardization of EM data set management and comparability of results across studies monitoring medication adherence with EM. The CleanADHdata.R script has broad applicability in digital health, as it can be used to clean adherence data collected through various digital technologies.

## Appendix

Multimedia Appendix 1 Description of the optional sheets to be filled in the Auxiliary Data Excel file.

Multimedia Appendix 2 Codebook of the cleaned electronic monitor data set “implementation.xlsx”.

Multimedia Appendix 3 Tutorial of the CleanADHdata.R script presented during the International Conference for Medication Adherence (ESPACOMP) in November 2022.

## Abbreviations

EM: electronic monitor
ESPACOMP: International Conference for Medication Adherence
MEMS: medication event monitoring system
OAT: oral anticancer treatment
OpTAT: optimizing targeted anticancer therapies

## Data Availability

The data sets generated and analyzed during this study are available at GitHub [23].
