# Supplementary material for: Using a Semiautomated Procedure (CleanADHdata.R Script) to Clean Electronic Adherence Monitoring Data: Tutorial
Source: JMIR Form Res. 2024 May 22;8:e51013. doi: 10.2196/51013 (PMC11153970; doi:10.2196/51013)
Supplement: Multimedia Appendix 1 [file formative_v8i1e51013_app1.docx]

| Optional variables in the Excel file | | | | | |
| --- | --- | --- | --- | --- | --- |
| Sheets | PatientCovariables | EMCovariables | AddedOpenings | NonMonitoredPeriods | AdverseEvents |
|  |  |  |  |  |  |
| Description of each variable as a header of a row to be filled in the required format | -PatientCode is the patient’s identifier.  -Start date is the start of a period for which a patient covariable applies (eg, the patient was included in several phases in a study).  -End date is the end of a period for which a patient covariable applies. | -PatientCode is the patient’s identifier.  -Monitor is the identifier of each EM used by the patient (1 EM per line).  -Start date is the start of a period for which an EM covariable applies.  -End date is the end of a period for which an EM covariable applies. | -PatientCode is the patient’s identifier.  -Monitor is the identifier of the EM for which an added or deleted opening is to be inserted at a specific date (1 EM per line).  -Date is the date during the monitoring on which a specific number of EM openings (indicated in AddedOpenings variable) is to be added.  -AddedOpenings (positive or negative number integer) is the number of EM openings that need to be added (+*x*), for example in case of a pocket dose, or deleted (–*x*), for example in case of an extra EM opening without intake, on the specific date indicated in the variable Date. | -PatientCode is the patient’s identifier.  -Monitor is the identifier of the EM for which a nonmonitored period was reported (1 EM per line).  -StartDate is the start of a period during which the EM was not used but treatment was taken (eg, in case of hospitalisation or holidays, with clear documentation).  -EndDate is the end of a period during which the EM was not used but treatment was taken. | -PatientCode is the patient’s identifier.  -Date is the date on which the adverse event was reported.  -AdverseEvent (any character string) is the description of the adverse event.  -AdverseEventGrade (any character string) is the grade of the adverse event, representing the severity of the adverse event (eg, grade 1=adverse effect bearable, grade 4=high toxicity). |
| Comments | Any relevant patient co-variable can be inserted by adding as many extra columns as needed (eg, presence of treatment discontinuation or censoring times according to the predefined definition, randomization group, gender, age, phase of a study). | Any relevant EM covariable can be inserted by adding as many extra columns as needed (eg, international nonproprietary name of the drug monitored, dose, number of tablets to be taken per EM opening). | This sheet allows amending the EM dataset based on patients’ EM use. To be noted, the number of pocket doses reported by the patients can be checked with pill count (ie, the difference between the number of pills delivered and returned between 2 consecutive pharmacy visits). | The implementation component of adherence is not calculated during nonmonitored periods and data are considered as missing during the analysis. | In this sheet, the adverse effect is added at a specific date. In case of several EM and drugs, the adverse effects are applied to the date of each EM, as it is assumed that the investigator cannot determine the causality between a drug and an adverse event. |
