# Supplementary material for: Using a Semiautomated Procedure (CleanADHdata.R Script) to Clean Electronic Adherence Monitoring Data: Tutorial
Source: JMIR Form Res. 2024 May 22;8:e51013. doi: 10.2196/51013 (PMC11153970; doi:10.2196/51013)
Supplement: Multimedia Appendix 2 [file formative_v8i1e51013_app2.docx]

| **By monitor** |
| --- |
| **PatientCode**: patient identification  **Monitor** : identification of the EM used by the patient  **Date**: date during the monitored period  **RecordedOpenings**: number of recorded EM opening(s) at each date, not corrected  **AddedOpenings**: number of EM openings added (+x) or deleted (-x) for each day  **CorrectedOpenings**: corrected number of EM openings according to the compilation of RecordedOpenings with the number of added or deleted EM openings –indicated in the variable “AddedOpenings”  **ExpectedOpenings:** number of EM expected openings at each date according to the drug regimen  **NonMonitored:** non-monitored periods are indicated by “TRUE” at each date they occurred.  **Implementation**: patient treatment implementation at each date for the considered EM.  **RelativeDate**: count of monitored days from the first to the last day of the monitoring period for each EM used.  **AdverseEvents**: adverse events reported at each day, separated by a comma. The corresponding grade for each adverse effect is added into brackets.  The next variables presented are the EM covariables from the AuxiliaryData file. |
| **By patient** |
| **PatientCode**: patient identification  **Date**: date during the monitored period  **MonitorsNb** : for each date, the number of EM for which data are collected to calculate patient implementation  **Implementation**: patient treatment implementation based on implementation for all EM used by the patient.  **RelativeDate**: count of monitored days from the day of the first EM used to the last day of the last EM used.  **AdverseEvents**: adverse events reported at each day, separated by a comma. The corresponding grade for each adverse effect is added into brackets.  The next variables presented are the patient covariables from the AuxiliaryData file. |
| **Summary by monitor** |
| **PatientCode**: patient identification  **Monitor**: identification of the EM used by the patient  **Implementation**: the implementation rate is calculated based on the number of days with an optimal implementation (=1) for the EM over the number of monitored days for each EM (i.e., non-monitored periods are excluded). |
| **Summary by patient** |
| **PatientCode**: patient identification  **Implementation**: the implementation rate is calculated based on the number of days with an optimal implementation for all EM used by the patient (=1) over the number of monitored days (i.e., non-monitored periods are excluded). |
