# Supplementary material for: Using a Semiautomated Procedure (CleanADHdata.R Script) to Clean Electronic Adherence Monitoring Data: Tutorial
Source: JMIR Form Res. 2024 May 22;8:e51013. doi: 10.2196/51013 (PMC11153970; doi:10.2196/51013)
Supplement: Multimedia Appendix 3 [file formative_v8i1e51013_app3.pdf]

A semi-automated procedure to clean and enrich electronic monitoring adherence data
The CleanADHdata.R script

ESPACOMP Adherence Data Analysis workshop
November 17th, 2022

Carole Bandiera, PhD student
Jérôme Pasquier, PhD, statistician
Isabella Locatelli, PhD, statistician
Marie P. Schneider, PhD

Aims of this presentation

- Introducing the context of the development of the CleanADHData.R script
- Presenting a tutorial to use the CleanADHData.R script
- Use of the script with simulated data
- Having your feedback about the use of the script

Context: need to clean electronic monitoring (EM) adherence database

90050	2016-06-11	1
90050	2016-06-11	1
90050	2016-06-12	1
90050	2016-06-12	1
90050	2016-06-13	0
90050	2016-06-13	0
90050	2016-06-14	0
90050	2016-06-14	0
90050	2016-06-15	0
90050	2016-06-15	0
90050	2016-06-16	0
90050	2016-06-16	0
90050	2016-06-17	0
90050	2016-06-17	1
90050	2016-06-18	1
90050	2016-06-25	1
90050	2016-06-25	1
90050	2016-06-26	0
90050	2016-06-26	0
90050	2016-06-27	1
90050	2016-06-27	1
90050	2016-06-28	0
90050	2016-06-28	0
90050	2016-06-29	1
90050	2016-06-29	1

Example of MEMS data for a patient treated with an oral anticancer therapy

Is it a **cyclic** regimen?

Did the oncologist ask the patient to stop the treatment because of **toxicity**?

Did the patient have to stop the treatment because of **radiotherapy**?

Non-adherence or adherence or treatment specific regimen?

Aim: developing a script in the statistical software R to **clean and enrich** MEMS data with information provided upon patient's report and medical record

Methods : CleanADHdata.R script cleans MEMS data

RAW data

PatientCode	Monitor	Date
90020	426785_1	08/08/2015 07:14:00
90020	426785_1	09/08/2015 07:35:00
90020	426785_1	10/08/2015 07:15:00
90020	426785_1	11/08/2015 06:18:00
90020	426785_1	12/08/2015 07:12:00
90020	426785_1	13/08/2015 07:10:00
90020	426785_1	14/08/2015 07:13:00
90020	426785_1	15/08/2015 07:19:00

+

Information

PatientCode	Monitor	ExpectedOpenings	StartDate	EndDate
90030	426783_1	2	27.10.2015	25.04.2016
90030	426784_1	1	27.10.2015	21.03.2016
90030	426784_1	0	22.03.2016	04.04.2016
90030	426784_1	1	05.04.2016	25.04.2016

PatientCode	Monitor	StartDate	EndDate	Comments
90030	426783_1	29.02.2016	03.03.2016	Hospitalisation
90030	426783_1	13.04.2016	21.04.2016	Hopitalisation

PatientCode	Monitor	Date	AddedOpenings	Comments
90050	426789_1	15.02.2016	-1	Opening but did not take
90050	426789_1	16.02.2016	1	Pocket dose
90050	426789_1	20.03.2016	-1	Pharmacy opening

CleanADHdata.R script*

```
# ---------------------- Import auxiliary data file ----------------------- #

# Select the auxiliary data file. The `eventslist` files must be in the same
# directory
#auxiliaryDataFile <- "C:/Users/cbandier/Desktop/2021.05.21_AuxiliaryData_OpTAT.xlsx"
auxiliaryDataFile <- file.path(
  "~/Projects/Consultations/Bandiera Carole (MEMS)/working-directory",
  "2021.06.02_AuxiliaryData_OpTAT_Test.xlsx"
)
#auxiliaryDataFile <- file.path(
#  "~/Projects/Consultations/Bandiera Carole (CovADH)/working-directory",
#  "Auxiliarly_data_COVADH_13.07.2021.xlsx"
#)
cap <- "Select the auxiliary data file"
if (!file.exists(auxiliaryDataFile)) {
  if (Sys.info()["sysname"] == "Windows") {
    auxiliaryDataFile <- choose.files(caption = cap, multi = FALSE)
  } else {
    auxiliaryDataFile <- tk_choose.files(caption = cap, multi = FALSE)
    auxiliaryDataFile <- paste(auxiliaryDataFile, collapse = " ")
  }
}
rm(cap)

# Define working directory (this is the directory which contains the auxiliary
# data file
workingDirectory <- dirname(auxiliaryDataFile)
```

Implementation sheet enriched, in an adapted format for analysis

PatientCode	Date	Monitor	ObservedOpenings	ExpectedOpenings	Implementation
90010	2015-07-24	426786_1	1	1	1
90010	2015-07-25	426786_1	2	2	1
90010	2015-07-26	426786_1	2	2	1
90010	2015-07-27	426786_1	2	2	1
90010	2015-07-28	426786_1	2	2	1
90010	2015-07-29	426786_1	2	2	1

Possibilities: calculation of **implementation** by monitor and by patient

*Implementation at each day=1 if the patient opens the MEMS **at least** the number of times it is expected, otherwise implementation=0

Methods: tutorial to use the CleanADHdata.R script

Go to https://github.com/jpasquier/CleanADHdata

data-simulated

❷ Simulated AuxiliaryData

❶ Simulated raw data

❸ Implementation.xlsx (database cleaned)

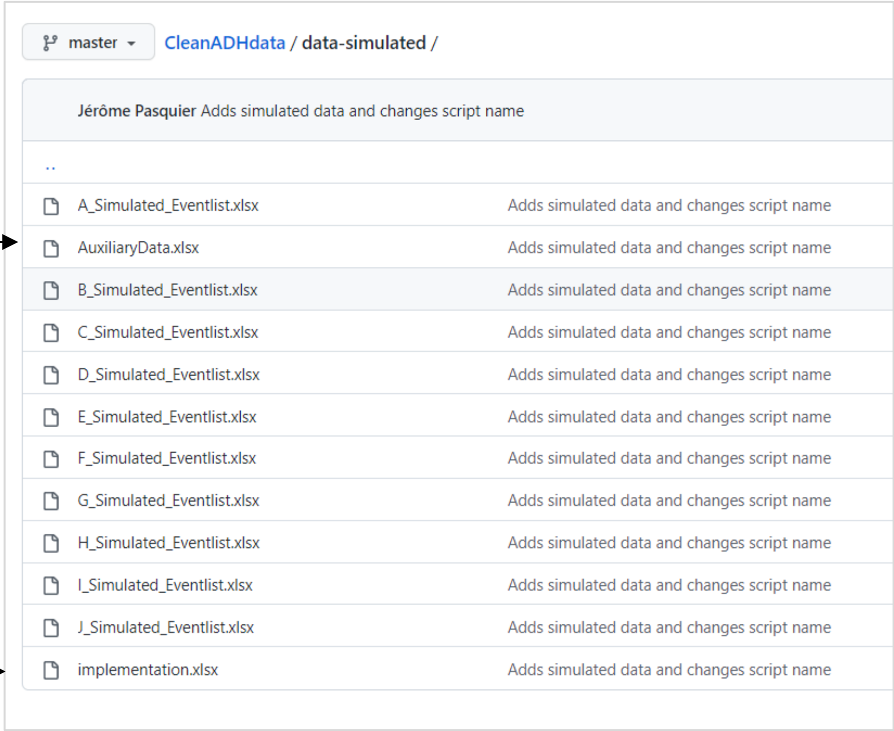

Methods: tutorial to use the CleanADHdata.R script (1/7)

1. Extraction of the EM raw data

↳ From the MEDAMIGO software AARDEX GROUP

↳ From an Excel sheet in the required format

The three columns are to be entitled "PatientCode", "Monitor" and "Date" in the first row

PatientCode	Monitor	Date
A	125A	2020-10-05 07:50:01 UTC
A	125A	2020-10-05 14:54:55 UTC
A	125A	2020-10-06 18:09:57 UTC

Methods: tutorial to use the CleanADHdata.R script (2/7)

2. Gathering the patients’ EM raw data **into the same file folder**

Filenames have to contain the **“eventslist”** or **“eventlist”** character string

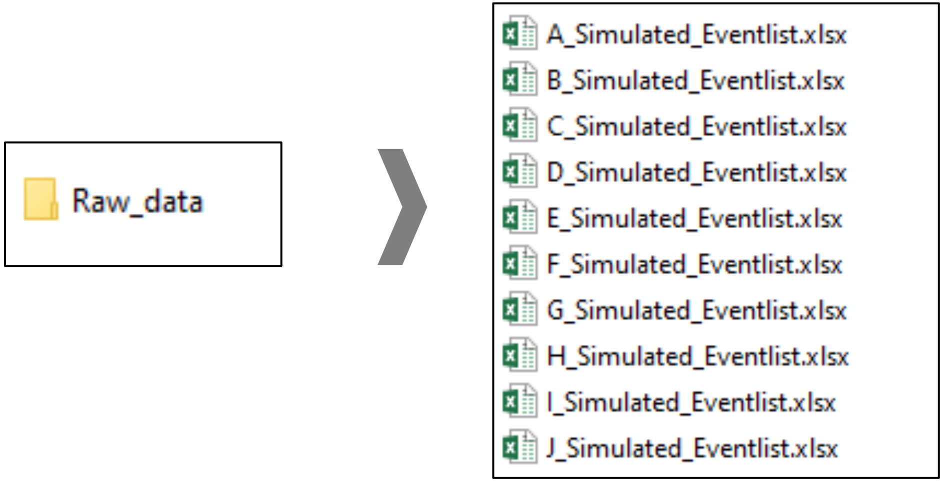

Methods: tutorial to use the CleanADHdata.R script (3/7)

3. Preparing the **Auxiliary data** Excel sheet

↳ Investigators need to determine in advance which relevant variables to collect (e.g., regimen changes, hospitalization dates, periods of EM non-use)

Composition of the Auxiliary Data Excel Sheet: 7 different sheets

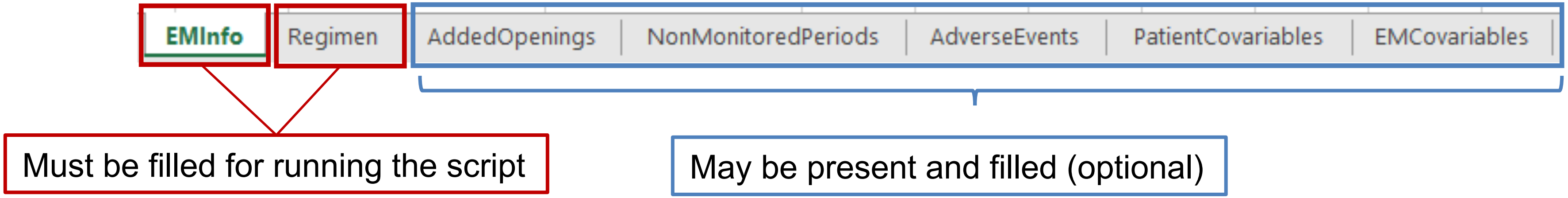

Methods: tutorial to use the CleanADHdata.R script

EMInfo

For each EM, start date of use

For each EM, end date of use

PatientCode	Monitor	StartDate	EndDate	Comment
A	125A	2020-10-05	2021-02-01	
B	78KL	2015-09-12	2016-03-17	
B	9863	2015-09-12	2016-03-03	
C	48PI	2020-03-22	2020-08-17	
C	L6OI	2020-03-22	2020-08-27	
D	789OPS	2015-09-12	2015-09-29	

Identifier of the patient

Identifier of each EM used by the patient (one EM per line)

Optional, may help you when gathering data (ignored by the script)

The cleaned database is truncated by the **start and end dates** of each EM use.

Methods: tutorial to use the CleanADHdata.R script

Regimen

Number of expected EM opening(s) per day based on treatment regimen prescription for the period of time specified between StartDate and EndDate

Start and end date of the period for which the number of daily expected opening applies

PatientCode	Monitor	ExpectedOpenings	StartDate	EndDate	On	Off
B	78KL	1	2015-09-12	2016-03-17		
B	9863	2	2015-09-12	2015-10-01		
B	9863	0	2015-10-02	2015-10-16		
B	9863	2	2015-10-17	2016-03-03		
C	48PI	1	2020-03-22	2020-07-18		

Identifier of the patient

Identifier of each EM used by the patient (one EM per line)

In case of a cyclic regimen, number of days during the ON and OFF phases (otherwise to be left empty)

Methods: tutorial to use the CleanADHdata.R script

AddedOpenings

Date during the monitoring on which a specific number of EM opening is to be added

The number of EM opening(s) that need to be added (+x) or deleted (-x), on the specific date indicated in the variable Date

PatientCode	Monitor	Date	AddedOpenings	Comments
B	78KL	2015-11-04	1	Pocket dose
B	78KL	2015-09-12	-1	PharmaOpening
B	78KL	2015-10-10	-1	PharmaOpening
B	78KL	2015-11-07	-1	PharmaOpening

Identifier of the patient

Identifier of the EM for which an added or deleted opening is to be inserted at a specific date (one EM per line)

Optional, may help you when gathering data (ignored by the script)

Methods: tutorial to use the CleanADHdata.R script

NonMonitoredPeriods

Identifier of the EM for which a non-monitored period was reported (one EM per line)

PatientCode	Monitor	StartDate	EndDate	Comments
B	78KL	2016-01-15	2016-01-18	Hospitalization
B	9863	2016-01-15	2016-01-18	EM not used (holidays)
B	78KL	2016-02-28	2016-03-07	Hospitalization
B	9863	2016-02-28	2016-03-03	Hospitalization
H	ASPJD7	2017-02-07	2017-02-16	Hospitalization

Identifier of the patient

the start and end of a period during which the EM was not used but treatment was taken

Optional, may help you when gathering data (ignored by the script)

The implementation component of adherence is not calculated during non-monitored periods and has to be considered as missing during the analyses.

Methods: tutorial to use the CleanADHdata.R script

AdverseEvents

Date on which the adverse event was reported

Grade of the adverse event (e.g., grade 0=adverse effect bearable, grade 4=high toxicity)

PatientCode	Date	AdverseEvent	AdverseEventGrade	Comments
B	2015-09-12	pain		
B	2015-10-08	mouth ulcers	1	
B	2015-10-10	fatigue	2	
C	2020-04-02	diarrhea	1	
C	2020-05-11	diarrhea	2	

Identifier of the patient

Description of the adverse event

Optional, may help you when gathering data (ignored by the script)

Methods: tutorial to use the CleanADHdata.R script

PatientCovariables

Any relevant patient co-variable can be inserted by adding as many extra columns as needed

PatientCode	StartDate	EndDate	Gender	Age	Discontinuation_censure
A	2020-10-05	2021-02-01	1	65	0
B	2015-09-12	2016-03-17	2	48	0
C	2020-03-22	2020-08-27	2	61	1

Identifier of the patient

start and end of a period for which a patient covariable applies

Methods: tutorial to use the CleanADHdata.R script

EMCovariables

Start and end of a period for which a EM covariable applies

Any relevant EM co-variable can be inserted by adding as many extra columns as needed

PatientCode	Monitor	StartDate	EndDate	Cyclic	DCI
A	125A	2020-10-05	2021-02-01	0	Pazopanib
B	78KL	2015-09-12	2016-03-17	0	Trametinib
B	9863	2015-09-12	2016-03-03	0	Dabrafenib
C	48PI	2020-03-22	2020-08-17	0	Trametinib

Identifier of the patient

Identifier of each EM used by the patient (one EM per line)

Methods: tutorial to use the CleanADHdata.R script (4/7)

4. Running the CleanADHData.R script (available on https://github.com/jpasquier/CleanADHdata/tree/master/R)

R

⇩

cleanADHdata.R

⇩

```r
#     ____ __                  ___   ___  __ __   __      __
#    / __// /__  ___ _ ___   / _ |  / _ \ / // /__/ /__ _ / /_ ___ _
#   / /__ / // -_)/ _ `// _ \ / __ | / // // _  // _  // _ `// __// _ `/
#   \___//_/ \__/ \_,_//_//_//_/ |_|/____//_//_/ \_,_/ \_,_/ \__/ \_,_/

# TODO:

# POSSIBLE OPTIONS:
# * Also read CSV files for raw data (in addition to Excel files))
# * Case-insensitive variable names

# ------------------------------ Libraries ------------------------------ #

# Install missing packages and load required libraries
pkgs <- c("readxl", "writexl")
npkgs <- pkgs[!(pkgs %in% installed.packages()[, "Package"])]
if(length(npkgs)) {
  install.packages(npkgs)
}
for (pkg in pkgs) {
  library(pkg, character.only = TRUE)
}
if (Sys.info()["sysname"] != "Windows") {
  if (!("tcltk" %in% installed.packages()[, "Package"])) {
    install.packages("tcltk")
  }
  library(tcltk)
}
rm(pkgs, npkgs, pkg)
```

- Open R

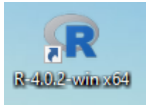

- Run the script

Methods: tutorial to use the CleanADHdata.R script

i) A window allows to select the AuxiliaryData Excel file

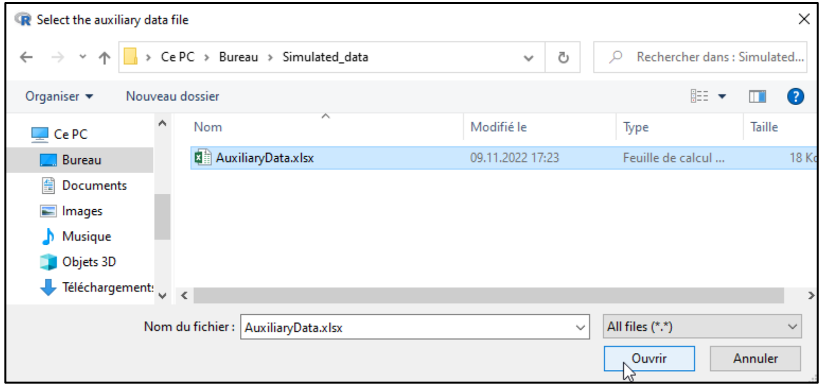

ii) A second window opens up to select the folder that contains the EM raw data

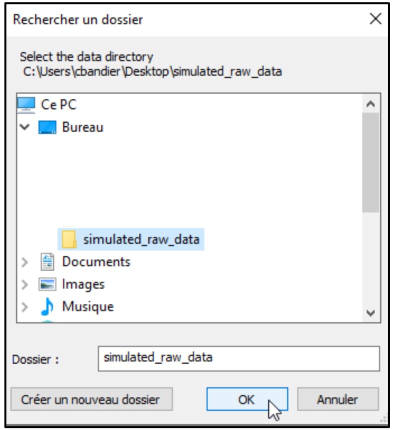

Methods: tutorial to use the CleanADHdata.R script (5/7)

5. After the end of processing, the R script generates:

- the cleaned and enriched EM dataset, called **"implementation.xlsx"** in the same file folder as the AuxiliaryData Excel sheet
- Logs file

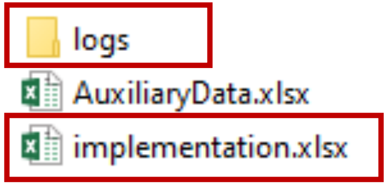

Methods: tutorial to use the CleanADHdata.R script (6/7)

6. Checking errors with the log files

- 2 log files are created, located in the same file folder as the AuxiliaryData Excel sheet

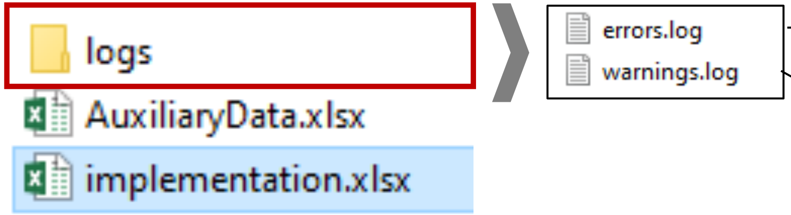

"errors" will show critical errors that affect implementation calculation

"warnings" indicates certain inconsistencies in the Auxiliary data file

After all errors or warnings are resolved, implementation.xlsx is ready for analysis

Methods: tutorial to use the CleanADHdata.R script (7/7)

7. Implementation.xlsx

The implementation rate is calculated **based on the number of days with an optimal implementation (=1)** for the EM over the number of monitored days for each EM (i.e., non-monitored periods are excluded)

The implementation rate is calculated based on the number of days with an optimal implementation for all EM used by the patient (=1) over the number of monitored days (i.e., non-monitored periods are excluded).

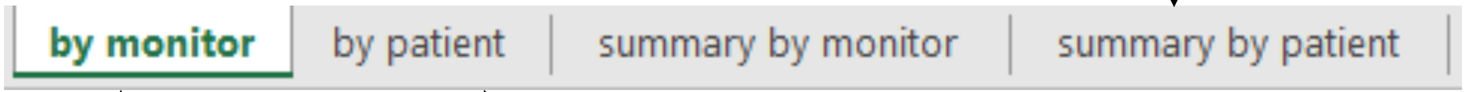

Medication implementation for the monitor at each day is considered

- optimal (=1) if the number of corrected EM openings is **at least equal** to the number of expected EM openings.
- suboptimal (=0) otherwise

Medication implementation for the patient is calculated at each day **by the product of the implementation outcome of each patient's EM**. In case of a sub-optimal implementation (=0) at a given day x <u>for at least one</u> EM monitored, the patient implementation at that day x will be suboptimal (=0).

Methods: tutorial to use the CleanADHdata.R script

Non-monitored period (empty cell)

by patient

PatientCode	Date	MonitorsNb	Implementation	RelativeDate	AdverseEvents	Gender	Age	Discontinuation_censure
A	2021-01-12	1	1	100		1	65	0
A	2021-01-13	1	1	101		1	65	0
A	2021-01-14	1		102		1	65	0
A	2021-01-15	1		103		1	65	0
A	2021-01-16	1		104		1	65	0
A	2021-01-17	1		105		1	65	0
A	2021-01-18	1	1	106		1	65	0
A	2021-01-19	1	1	107		1	65	0
A	2021-01-20	1	1	108		1	65	0
A	2021-01-21	1	1	109		1	65	0
A	2021-01-22	1	1	110		1	65	0
A	2021-01-23	1	1	111		1	65	0
A	2021-01-24	1	1	112		1	65	0
A	2021-01-25	1	1	113		1	65	0
A	2021-01-26	1	1	114	fatigue (1)	1	65	0
A	2021-01-27	1	1	115		1	65	0
A	2021-01-28	1	1	116		1	65	0
A	2021-01-29	1	1	117		1	65	0
A	2021-01-30	1	1	118		1	65	0
A	2021-01-31	1	1	119		1	65	0
A	2021-02-01	1	1	120		1	65	0
B	2015-09-12	2	1	1	pain	2	48	0
B	2015-09-13	2	1	2		2	48	0
B	2015-09-14	2	0	3		2	48	0
B	2015-09-15	2	1	4		2	48	0
B	2015-09-16	2	0	5		2	48	0
B	2015-09-17	2	1	6		2	48	0

Non implementation

Cleaned EM database ready for analysis

Methods: tutorial to use the CleanADHdata.R script

summary by monitor

A first overview of the results before analysis

PatientCode	Monitor	Implementation
A	125A	0.99
C	48PI	0.97
F	7374WOS	0.97
E	74RPZ	0.98
D	789OPS	1.00
B	78KL	0.86
B	9863	0.97
H	ASPJD7	0.96
G	AU7	0.98
H	J878D	0.93
C	L6OI	0.99
J	OJ5	0.99
I	S7	0.97
I	SOD7	0.97
J	SZU12	0.98

⇨

Patient B implemented less Trametinib (monitor 78KL) compared to Dabrafenib (monitor 9863)

Methods: tutorial to use the CleanADHdata.R script

summary by patient

A first overview of the results before analysis

PatientCode	Implementation
A	0.99
B	0.84
C	0.95
D	1.00
E	0.98
F	0.97
G	0.98
H	0.89
I	0.97
J	0.97

Patient B has the lowest implementation

Patient D implemented the treatment optimally

Discussion

- Based on the cleaned and enriched dataset we can evaluate **implementation, persistence and adherence analysis** (e.g., with Generalized Estimating Equations), including covariates

- The **calculation of medication implementation** can be adapted directly in the script under the section "implementation calculation" (line 736)

```
# ---------------------- Implementation calculation ------------------------ #
```

- A **tutorial** will be submitted for publication to guide future users of the script

➢ Would you use the CleanADHdata.R script?
➢ Would you have any inputs?

Thank you for listening

Questions?

carole.bandiera@unige.ch
